# Repurposing Potential of Riluzole as an ITAF Inhibitor in mTOR Therapy Resistant Glioblastoma

**DOI:** 10.3390/ijms21010344

**Published:** 2020-01-05

**Authors:** Angelica Benavides-Serrato, Jacquelyn T. Saunders, Brent Holmes, Robert N. Nishimura, Alan Lichtenstein, Joseph Gera

**Affiliations:** 1 Department of Research & Development, Greater Los Angeles Veterans Affairs Healthcare System, Los Angeles, CA 91343, USA; 2 Department of Neurology, David Geffen School of Medicine at UCLA, Los Angeles, CA 90095, USA; 3 Jonnson Comprehensive Cancer Center, University of California-Los Angeles, Los Angeles, CA 90095, USA; 4 Department of Medicine, David Geffen School of Medicine at UCLA, Los Angeles, CA 90095, USA; 5 Molecular Biology Institute, University of California-Los Angeles, Los Angeles, CA 90095, USA

**Keywords:** riluzole, hnRNP A1, ITAF, mTOR, drug resistance, glioblastoma

## Abstract

Internal ribosome entry site (IRES)-mediated protein synthesis has been demonstrated to play an important role in resistance to mechanistic target of rapamycin (mTOR) targeted therapies. Previously, we have demonstrated that the IRES *trans*-acting factor (ITAF), hnRNP A1 is required to promote IRES activity and small molecule inhibitors which bind specifically to this ITAF and curtail IRES activity, leading to mTOR inhibitor sensitivity. Here we report the identification of riluzole (Rilutek^®^), an FDA-approved drug for amyotrophic lateral sclerosis (ALS), via an *in silico* docking analysis of FDA-approved compounds, as an inhibitor of hnRNP A1. In a riluzole-bead coupled binding assay and in surface plasmon resonance imaging analyses, riluzole was found to directly bind to hnRNP A1 and inhibited IRES activity via effects on ITAF/RNA-binding. Riluzole also demonstrated synergistic anti-glioblastoma (GBM) affects with mTOR inhibitors in vitro and in GBM xenografts in mice. These data suggest that repurposing riluzole, used in conjunction with mTOR inhibitors, may serve as an effective therapeutic option in glioblastoma.

## 1. Introduction

Despite recent advances in cancer drug discovery, including high-throughput screening and structure-based drug design, significant increases in the number of new approved anticancer drugs which progress to the clinic is lacking [1]. Moreover, the timeframe required for single drug development on average has increased from 7.9 years to 13.9 years, at an average cost of bringing to market of ~1.8 billion US dollars [1,2]. Thus, identifying current drugs for novel antitumor indications is a promising strategy to accelerate drug development.

GBM is a particularly lethal tumor of the CNS which in part is due to the difficulty in complete surgical resection and the eventual development of resistance to drug therapies [3]. The median survival for patients diagnosed with this tumor remains a dismal twelve months [4]. Hyperactivation of the PI3K pathway is observed in approximately 90% of all GBMs as a result of EGFR amplification or activating mutations and/or PTEN loss [5,6,7]. This leads to durable activation of the downstream effector, the mechanistic target of rapamycin (mTOR) kinases [8,9]. mTOR is present in cells as part of two distinct kinase complexes, mTORC1 and mTORC2 each with distinct substrate specificities [10].

Allosteric mTORC1 inhibitors such as rapamycin and several rapalogs have been unsuccessful as monotherapies in the clinic for GBM as a result of loss of feedback regulation driving AKT activation [11]. Moreover, mTORC2 has been demonstrated to mediate GBM growth, mobility, invasion and drug resistance [12]. Recently, an mTORC2-specific inhibitor was developed which has demonstrated significant anti-GBM activity in pre-clinical experiments [13]. Thus, these lines of investigations support the continued therapeutic development of effective mTOR inhibitors for GBM [14].

The direct signaling relationship between the mTORC1 and mTORC2 complexes suggests that several mechanisms of mTOR inhibitor resistance may play a role in intrinsic resistance to this class of compounds [15,16,17]. Our previous studies have shown that both allosteric and direct mTOR kinase inhibitors are able to induce a transcript-specific protein synthesis salvage pathway, which is able to enhance the IRES-dependent translation of critical mRNAs required for cell-cycle transit leading to resistance to mTOR inhibitors [16,18,19,20]. IRES-dependent translation has been implicated in, and thought to play a major contributory role in tumor growth, survival and chemoresistance [21,22,23]. Recently, we identified a class of small molecule inhibitors which blocks the ability of the ITAF hnRNP A1 from associating with both of the cyclin D1 and c-myc IRES RNAs leading to reduced translation of these determinants and resulting in mTOR inhibitor sensitivity of GBM cells [24]. 

In this study, we continue these efforts and report the identification of riluzole as an inhibitor of hnRNP A1 ITAF activity via an *in silico* screen of FDA-approved compounds. We demonstrate that riluzole inhibits IRES-dependent translation and blocks hnRNP A1 binding to both the cyclin D1 and c-myc IRESs resulting in markedly reduced translational efficiencies of these transcripts. We further show that riluzole directly binds hnRNP A1 in SPR experiments and a riluzole coupled bead pull-down assay. Additionally, an hnRNP A1 mutant in which critical inhibitor interacting residues comprising the binding pocket were altered resulting in the inability of these compounds to bind hnRNP A1, was also unable to bind riluzole. Finally, co-treatment with riluzole and PP242 results in synergistic anti-GBM affects in vitro and in xenograft experiments.

## 2. Results

### 2.1. Molecular Docking Screening Identifies Riluzole as a Potential hnRNP A1 Inhibitor

**Figure 1:**
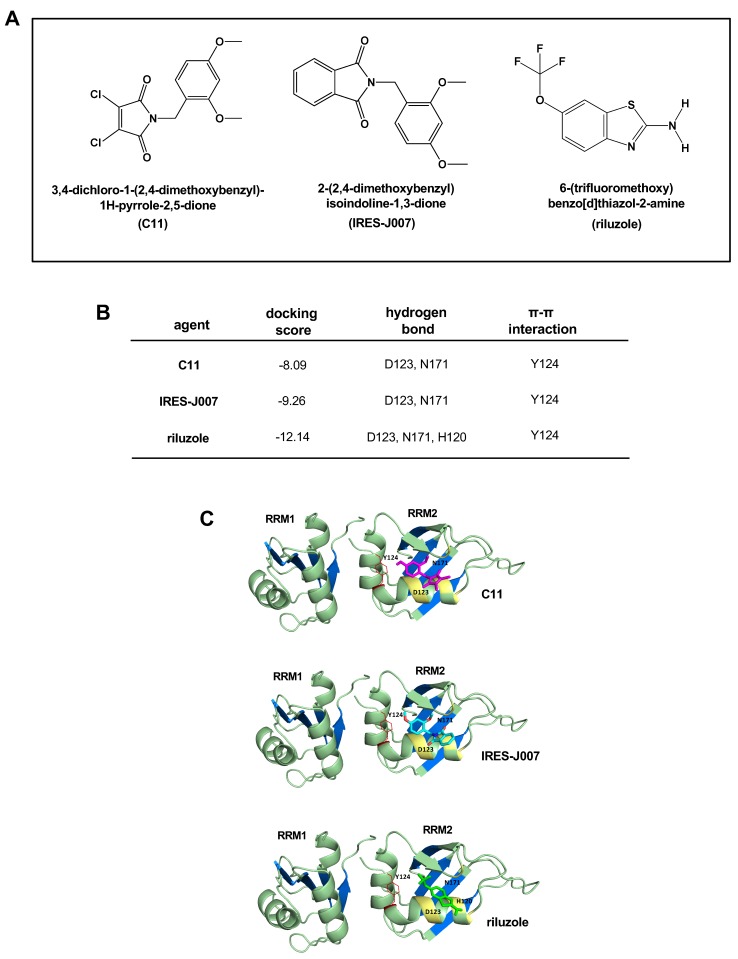
Riluzole is an hnRNP A1 inhibitor identified via *in silico* molecular docking analyses. (**A**) Chemical structures of hnRNP A1 inhibitors. (**B**) Interaction properties of compounds C11, IRES-J007 and riluzole. Critical residues of hnRNP A1 for inhibitor binding are listed. (**C**) Conformers of C11 (magenta), IRES-J007 (cyan) and riluzole (chartreuse) with the lowest binding free energies bound to the inhibitor-binding site of human hnRNP A1 (UP1 fragment, 1HA1 model) with labeled residues. The domain representation of the UP1 crystal structure is shown in green with RNP residues of RRM1 and RRM2 labeled in blue.

In this study, we utilized a molecular docking strategy to identify potential inhibitors which were predicted to bind to the ITAF hnRNP A1. Previously, we had identified a class of inhibitors via a yeast three-hybrid screen in which the tool compound C11, shown in Figure 1A, bound to a small pocket structure close to RRM2 within the UP1 fragment of hnRNP A1 [24]. C11 was subsequently used in structure-activity relationship studies to derive an improved active analog IRES-J007. To generate unbiased predictive virtual docking models, we obtained the crystal structure of monomeric UP1 from the Protein Data Bank (PDB) and performed docking simulations using AutoDock Vina molecular modeling software [25]. We screened a ligand library from an FDA-approved drug database of 1500 compounds using a grid box (20 Å × 17 Å × 17 Å) encompassing the C11 and IRES-J007 inhibitor binding cavity of UP1. The binding modes were clustered based on the root-mean square deviation (RMSD) between the Cartesian coordinates of the ligand atoms. The docking results were then ranked by the binding free energy (see supplementary Table S1) and then the top 10 candidates filtered as potential hnRNP A1 inhibitors (summarized in supplementary Table S2). We subsequently tested these 10 candidates for their ability to affect basal cyclin D1 or c-myc IRES activity in 293T cells which express high levels of hnRNP A1 and show elevated IRES activity [24]. As shown in supplementary Table S2 the benzothiazole CNS compound, riluzole was the most effective inhibitor of IRES activity, markedly blocking both cyclin D1 and c-myc IRES activity and was chosen for further study. The docking scores of C11, IRES-J007 and riluzole all suggested high binding affinities in the inhibitor binding site of the PDB 1HA1 model (Figure 1B,C). On the basis of the docking simulations, C11 and IRES-J007 bound to D123 and N171 directly through hydrogen bonds and also formed a π-π interaction with Y124. Similarly, riluzole was predicted to interact with D123 and N171 via hydrogen bonding and the π-π interaction with Y124; however additionally with H120 via hydrogen bonding.

### 2.2. Riluzole Blocks IRES Activity and hnRNP A1-IRES mRNA Binding in Glioblastoma

**Figure 2:**
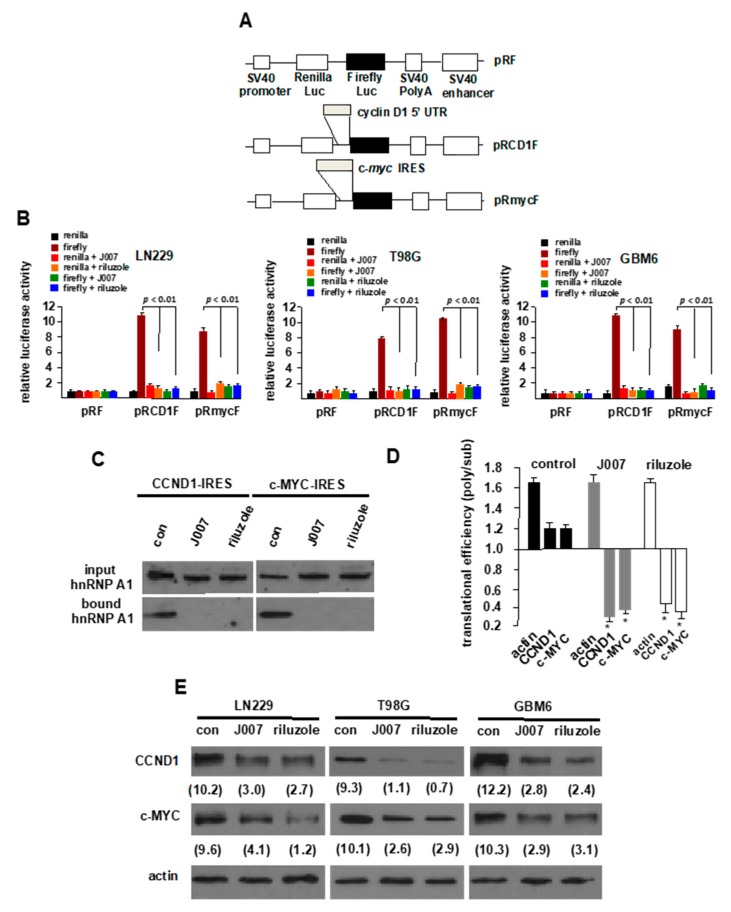
Riluzole inhibits IRES-mediated mRNA translation in GBM. (**A**) Schematic diagram of IRES reporter constructs used in this study. Constructs used are pRF, pRCD1F, containing the human cyclin D1 IRES, and pRmycF, containing the human c-myc IRES. (**B**) Relative *Renilla* and firefly luciferase activities obtained from established LN229, T98G GBM lines or short-term PDX line GBM6 transfected with the indicated constructs in the absence or presence of the inhibitor J007 (100 nM) or riluzole (2 µM). The mean and +SD are shown for three independent experiments. (**C**) RNA-pull down assays utilizing biotinylated cyclin D1 or c-MYC IRES RNAs. Cytoplasmic extracts of LN229 cells treated with J007 (100 nM) or riluzole (2 µM) as indicated were incubated with biotinylated cyclin D1 or c-MYC IRES RNAs and precipitated with streptavidin-Sepharose beads. Input and bound fractions were analyzed by immunoblotting using hnRNP A1 antibodies. Data shown are representative of experiments repeated two times. (**D**) Histogram showing translational efficiency of cyclin D1, c-MYC or actin mRNAs from LN229 cells treated with the indicated inhibitors as determined by qrt-PCR as the ratio between polysomal and non-polysomal/monosomal RNA fractions. qrt-PCR measurements were performed in quadruplicate and the mean and +S.D. are shown. (* *p* < 0.05, significantly different from CCND1 and c-MYC controls and CCND1 + J007, CCND1 + riluzole or c-MYC + J007 and c-MYC + riluzole). (**E**) Cyclin D1 and c-MYC protein levels in LN229, T98G or GBM6 cells treated with J007 (100 nM) or riluzole (2 µM) for 24 h. Immunoblots were quantified via densitometry and relative intensity values shown.

To further validate and explore the inhibitory effects of riluzole on hnRNP A1-mediated ITAF activity in other lines, we determined the effects on dicistronic IRES mRNA reporter activity in the GBM cell lines LN229 and T98G, as well as a short-term PDX GBM line, GBM6. We transiently transfected the constructs shown in Figure 2A, into the indicated cells and determined *Renilla* and firefly luciferase activities (readouts of cap-dependent and IRES initiation, respectively) following the indicated treatments with IRES-J007 or riluzole. As shown in Figure 2B, IRES-J007 and riluzole markedly inhibited both cyclin D1 and c-myc IRES activity consistent with the requirement of hnRNP A1 for IRES activity in these lines [20]. To determine whether riluzole would affect hnRNP A1 binding to the cyclin D1 or c-myc IRESs, we utilized LN229 cell extracts from cells treated with riluzole in RNA-pull down experiments. As shown in Figure 2C, hnRNP A1 bound effectively to both the cyclin D1 and c-myc IRES RNA sequences in control reactions; however in extracts from cell treated with either IRES-J007 or riluzole binding of hnRNP A1 was blocked. We further examined the translational state of the cyclin D1 and c-myc mRNAs in cells treated with the inhibitors via polysome analyses and as shown in Figure 2D, the translational efficiency of the cyclin D1 and c-myc transcripts was significantly reduced from cells treated with IRES-J007 or riluzole. Both cyclin D1 and c-myc mRNAs were shifted to nonpolysomal fractions indicating relatively poor translation while actin mRNAs whose translation initiation is exclusively cap-dependent was unaffected. We also noted that neither IRES-J007 or riluzole appeared to alter cyclin D1 or c-myc steady-state mRNA levels as total nonpolysomal plus polysomal mRNA content was unchanged as compared controls suggesting that the inhibitors do not affect transcription or mRNA stability. Finally, cyclin D1 and c-MYC protein levels in LN229, T98G and the patient-derived line GBM6 were markedly reduced (Figure 2E) following exposure to either IRES-J007 or riluzole. These data demonstrate that riluzole effectively inhibits cyclin D1 and c-myc IRES activity leading to reduced protein levels.

### 2.3. Riluzole Blocks Association of UP1 with IRES RNAs

**Figure 3:**
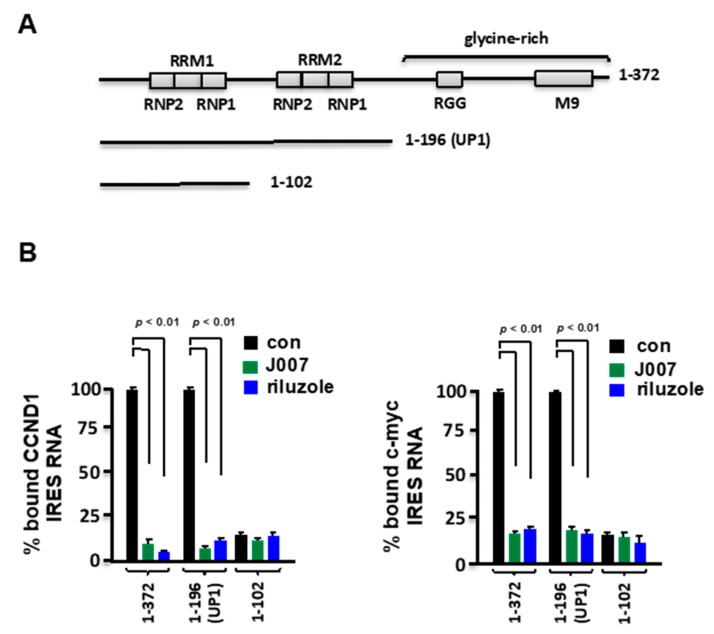
Riluzole-mediated inhibition of hnRNP A1 ITAF/IRES-binding requires only the UP1 fragment. (**A**) Schematic representation of GST-tagged hnRNP A1 deletion mutants. Mutant 1-196 constitutes the UP1 fragment of full-length human hnRNP A1. In the ∆130–158 mutant, the sequences encompassing RRM2 have been deleted. (**B**) Binding of either cyclin D1 (left panel) or c-MYC (right panel) IRES RNAs to GST-tagged hnRNP A1 mutants in the absence or presence of C11 or IRES-J007 as assayed by filter binding. Mean and +SD are shown for three independent experiments.

Previously, we demonstrated that the UP1 fragment of hnRNP A1 was sufficient to recapitulate C11 or IRES-J007-mediated inhibition of native hnRNP A1 binding to cyclin D1 or c-myc IRESs [24]. To determine whether riluzole would also similarly inhibit hnRNP A1 binding we tested the ability of IRES-J007 or riluzole to inhibit the association of full-length, as well as the deletion mutants shown in Figure 3A with either the cyclin D1 or c-myc IRESs as determined by filter binding assays. As shown in Figure 3B, The UP1 fragment (a.a. 1–196) containing RRM1 and RRM2 and immediately adjacent sequences effectively bound either of the IRES RNAs and binding was inhibitable by J007 or riluzole. A mutant encompassing the N-terminal 102 amino acids of hnRNP A1 did not demonstrate IRES binding. As the UP1 fragment contains the inhibitor binding pocket for IRES-J007 these data support the notion that riluzole inhibits IRES RNA binding by a similar mechanism as C11 and IRES-J007.

### 2.4. Riluzole Directly Binds to hnRNP A1 within the Predicted IRES-J007 Binding Pocket

**Figure 4:**
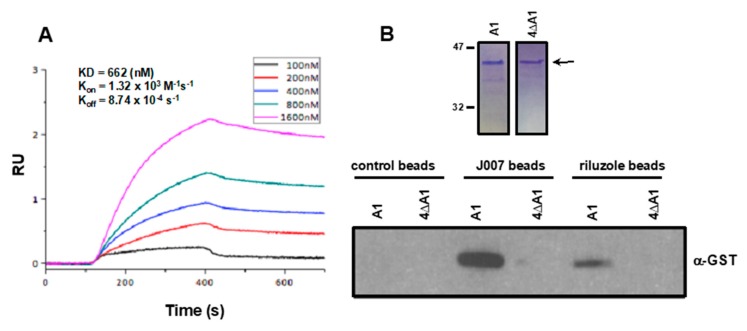
Riluzole interacts directly with hnRNP A1. (**A**) Surface plasmon resonance imaging monitoring the interaction of immobilized hnRNP A1 with riluzole. The KD, K_on_ and K_off_ were calculated by simultaneous non-linear regression using a 1:1 binding model and BIAevaluation 4.1 software. (**B**) Purified GST-tagged native and mutant A1 (4∆A1) hnRNP A1 harboring alanine substitutions at all four critical residues within the hnRNP A1 binding pocket (H120, D123, Y124 and N171) were added to uncross-linked, IRES-J007 and riluzole-crosslinked beads. Native and mutant (4∆A1) were resolved by SDS-PAGE and stained with GelCode™ Blue stain to assess purity (top panel). Binding of native hnRNP A1 to control, IRES-J007 and riluzole coupled beads was detected by immunoblotting with α-GST antibody (bottom panel). Data shown are representative of experiments repeated twice.

To further examine the mechanism by which riluzole inhibits hnRNP A1 we performed SPRi analyses of riluzole binding to immobilized hnRNP A1. As shown in Figure 4A, riluzole bound hnRNP A1 in a concentration-dependent manner and reached equilibrium rapidly. The K_d_ was determined from steady-state binding associations and was calculated at 662 nM supporting a direct interaction between riluzole and hnRNP A1. Previously, we generated a quadruple alanine substitution mutant of hnRNP A1 in which all four of the predicted interacting residues were mutated and this mutant failed to bind C11 or IRES-J007 in an in vitro inhibitor-bead coupled pull-down assay [24]. To further investigate whether riluzole bound to the same binding pocket and confirm the accuracy of our binding models we determined if riluzole could bind native versus the quadruple alanine substitution mutant of hnRNP A1 (4∆A1) in this assay. Recombinant GST-tagged native and mutant proteins were purified by glutathione affinity procedures and the purity was confirmed by SDS-PAGE followed by staining (Figure 4B, top panel). As shown in Figure 4B (bottom panel), purified proteins were subsequently incubated with control, IRES-J007 or riluzole beads and binding assessed by immunoblotting using α-GST antibodies. IRES-J007 or riluzole coupled beads bound native hnRNP A1, whereas control beads did not. Moreover, native hnRNP A1 bound specifically to IRES-J007 or riluzole linked beads while the quadruple alanine substitution mutant 4∆A1 did not associate with either of the inhibitor linked beads. These results suggest that riluzole binds hnRNP A1 through the residues predicted by the interaction model.

### 2.5. Riluzole and mTOR Inhibitors Display Synergistic Anti-GBM Properties In Vitro

**Figure 5:**
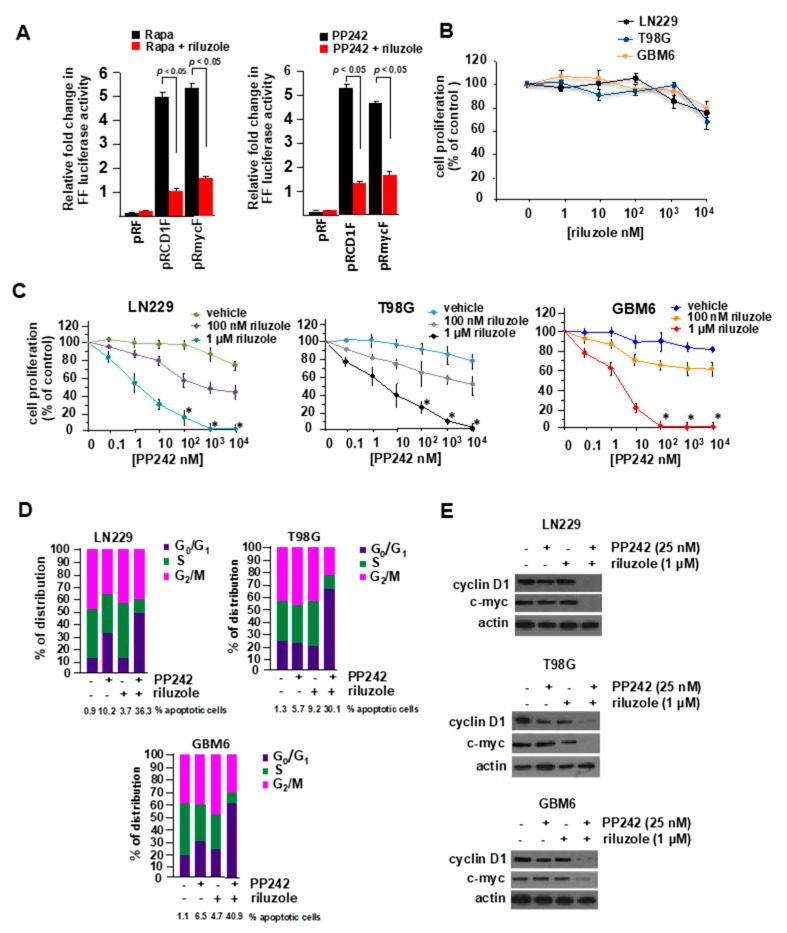
Synergistic anti-GBM effects of riluzole and mTOR inhibition. (**A**) Inhibition of mTOR inhibitor-induced IRES activity by riluzole. LN229 cells were transiently transfected with the indicated IRES mRNA reporter constructs and treated with either rapamycin (25 nM) alone or in conjunction with riluzole (1 µM) (left panel), or PP242 (25 nM) alone or PP242 plus riluzole (1 µM) (right panel) for 24 h. Luciferase activities were subsequently determined and results expressed as relative fold change in firefly (FF) luciferase activity. The mean and +S.D. are shown (*n* = 3). (**B**) Effects of riluzole on GBM cell line proliferation following exposure at 48 h. Mean ±S.D.; *n* = 3. (**C**) Effects of riluzole and PP242 cotreatments on GBM cell line proliferation. LN229, T98G or GBM6 cells were treated with increasing concentrations of PP242 alone or in combination with the indicated doses of riluzole for 48 h and cell proliferation relative to control treatments was determined via Cell Titer-Glo^®^ luminescent cell assays. Mean ± S.D., *n* = 3. (**D**) Cell-cycle phase distributions were assessed on LN229, T98G and GBM6 cells in the presence or absence of PP242 or riluzole as shown. The percentage of apoptotic cells is shown below each graph as determined via annexin V-FITC staining. (**E**) Immunoblots of cyclin D1, c-myc and actin levels from lysates of LN229, T98G or GBM6 cells treated with the indicated inhibitors at 24 h.

Several studies have demonstrated that mTOR inhibition induces upregulation of IRES activity as an intrinsic mechanism of resistance to these drugs [26,27,28]. Thus, we were interested in whether riluzole would potentiate PP242 cytotoxicity in GBM. Initially, we treated LN229 GBM cells with rapamycin (mTORC1 inhibitor) or PP242 (mTORC1/2 inhibitor) and determined whether riluzole would lead to an inhibition of IRES activity. As shown in Figure 5A (left panel), the combination of riluzole with rapamycin markedly inhibited both cyclin D1 and c-myc IRES activity relative to the induction of IRES activity observed in cells treated with rapamycin alone. Similarly, riluzole significantly blocked PP242-induced cyclin D1 and c-myc IRES activity (Figure 5A, right panel). We subsequently tested the effects of riluzole alone on GBM cell line proliferation. As shown in Figure 5B, no significant inhibition of cell proliferation was observed at any of the concentrations tested up to 10 µM. In the GBM cell lines, LN229, T98G and in the short-term patient derived line GBM6, combination treatments with riluzole at 100 nM and 1 µM over a wide range of PP242 concentrations (Figure 5C) resulted in synergistic inhibition of cell proliferation (CI = 0.45 at ED_50_ of 1:100). We then tested whether the combination of riluzole and PP242 would induce G_1_ arrest and apoptosis. As shown in Figure 5D, PP242 exposure increased G_1_ arrest and cotreatment with riluzole potentiated the effects of PP242. Additionally, PP242 induced apoptosis which was further enhanced by cotreatment with riluzole. Protein levels of cyclin D1 and c-MYC were also significantly reduced by the cotreatment of PP242 and riluzole relative to single agent treatments as shown in Figure 5E. These data demonstrate that cotreatments with PP242 and riluzole result in enhanced levels of G_1_ arrest and apoptosis in GBM cells.

### 2.6. In Vivo Effects of PP242 and Riluzole Cotherapy

**Figure 6:**
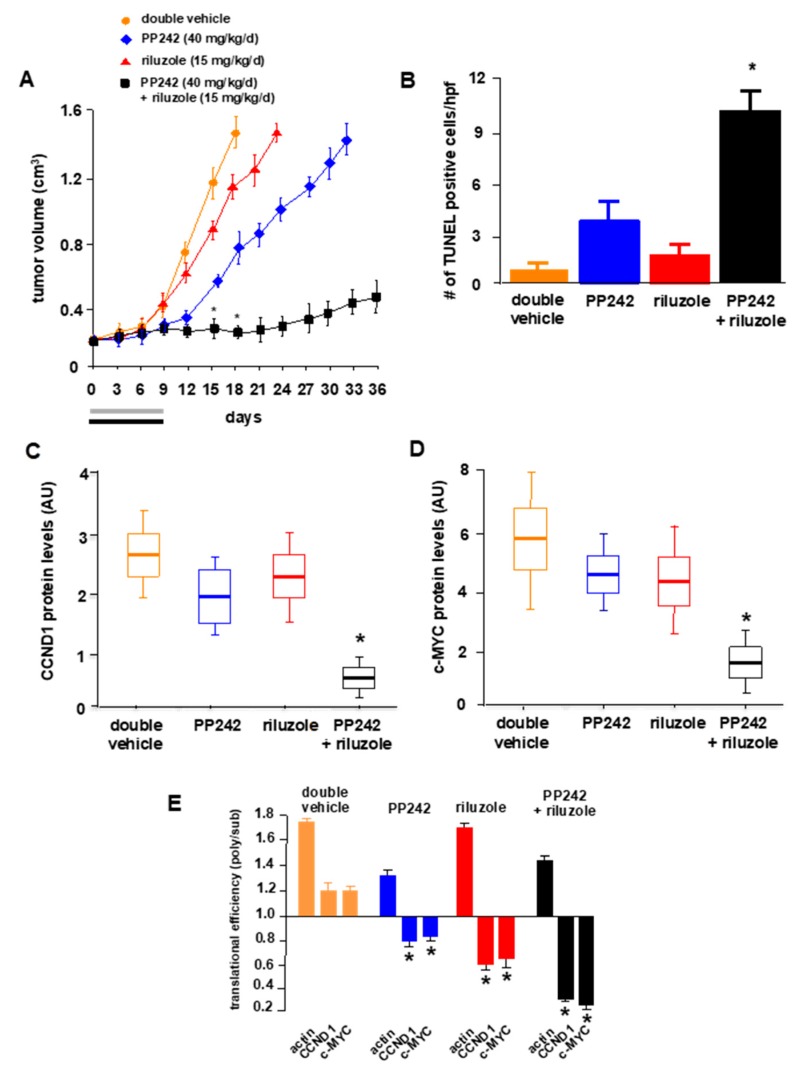
*In vivo* effects of riluzole and PP242 co-therapy. (**A**) Tumor volume of subcutaneous LN229 cell xenografts in mice treated with the indicated schedules of double vehicle, PP242 (grey bar), riluzole (black bar) or PP242 + riluzole for nine consecutive days and tumor growth monitored every 3 days following the start of treatment on day 0. * *p* < 0.05, significantly different from double vehicle, *n* = 6 mice per treatment group. (**B**) Sections prepared from harvested tumors at day 15 following the initiation of treatment schedules were subjected to TUNEL staining to determine apoptotic cell numbers. Data are expressed as the number of positive apoptotic bodies divided by high power field (hpf; 10–12 hpf/tumor). Values are means +S.D., * *p* < 0.05. (**C**) Box plots showing cyclin D1 protein levels in harvested tumors from mice treated with the indicated regimens. * *p* < 0.05. (**D**) As in (**C**), but values for c-MYC protein levels are shown. (**E**) Histogram showing translational efficiency of cyclin D1, c-myc or actin mRNAs determined by qrt-PCR as the ratio between polysomal and non-polysomal/monosomal RNA fractions at day 15. qrt-PCR measurements were performed in quadruplicate and the mean and +S.D. are shown. (* *p* < 0.05, significantly different from CCND1 and c-MYC double vehicle controls).

We conducted mouse xenograft studies to assess whether the combination of riluzole and mTOR inhibition would lead to significant anti-GBM affects in vivo. LN229 cells were implanted subcutaneously in SCID mice and once tumors grew to ~200 mm^3^ in size, mice were randomized and placed into treatment groups receiving double vehicle, PP242 (40 mg/kg/day), riluzole (15 mg/kg/day) or PP242 (40 mg/kg/day) plus riluzole (15 mg/kg/day). As shown in Figure 6A, tumor-bearing mice receiving monotherapy with PP242 resulted in significant inhibition of tumor growth rate (49% inhibition at day 15 following the initiation of treatment; tumor growth delay 4.0 days). Mice receiving monotherapy with riluzole alone at the dosing regime used did not exhibit a significant reduction in tumor volume as compared to mice receiving double vehicle which was consistent with the lack of effect of riluzole alone in cell culture experiments (see Figure 5B). The combination of PP242 and riluzole was significantly more effective than either of the monotherapies (95% inhibition at day 15; tumor growth delay, 21.5 days). We also observed that the mice tolerated this dosing regimen well without significant short or long-term toxicity or weight loss. We monitored the induction of apoptosis via TUNEL staining of sections from harvested tumors at autopsy. As shown in Figure 6B, significant increases in the numbers of TUNEL positive cells were present in tumors from mice treated with the combination of PP242 and riluzole (see also supplementary Figure S1). These data were supported by the observed increases in apoptotic cell numbers in vitro (see Figure 5D). The protein levels of cyclin D1 and c-MYC (Figure 6C,D) were determined from harvested tumors and mice receiving combination therapy displayed significantly reduced expression levels relative to either monotherapy. Finally, we examined the mRNA translational state of the cyclin D1 and c-myc transcripts from harvested tumors via polysome analysis as before. As shown in Figure 6E, tumors from mice receiving double vehicle treatments exhibited a cyclin D1 and c-myc polysomal to nonpolysomal/monosomal ratio of 1.19 and 1.20, respectively. Mice receiving PP242 monotherapy displayed significantly reduced polysome to nonpolysomal/monosomal ratios of cyclin D1 and c-myc mRNAs to 0.59 and 0.62, respectively. Actin mRNA polysomal distribution, whose synthesis is mediated via eIF-4E dependent initiation was markedly redistributed to nonpolysomal/monosomal fractions indicating effective inhibition of cap-dependent initiation. Consistent with the results observed of inhibiting IRES-mediated translation in vitro (see Figure 2D), riluzole treatment also reduced the translational efficiency of both cyclin D1 and c-myc mRNAs in tumors from mice, whereas actin mRNA translation was unaffected. Tumors from mice receiving PP242 and riluzole cotherapy displayed a larger reduction in cyclin D1 and c-myc mRNA translational efficiency compared to either monotherapy. These data suggest that riluzole inhibits cyclin D1 and c-myc IRES-mediated translation and further support the notion that the cyclin D1 and c-myc mRNAs are able to initiate translation via cap-dependent and IRES-dependent mechanisms.

## 3. Discussion

The ability to repurpose current drugs potentially affords an opportunity to find new indications for existing drugs in an efficient manner with reduced cost and risk. Our previous studies identified a small-molecule inhibitor which effectively blocked hnRNP A1-mediated IRES activity and docking studies in conjunction with mutational analysis of hnRNP A1 identified the binding site of this inhibitor [24]. In this study, we utilized models of the binding pocket structure to screen for compounds which were able to potentially occupy this site from a library of FDA-approved agents. The blood brain barrier penetrant compound riluzole demonstrated significant inhibition of IRES activity and synergistic anti-GBM affects with mTOR inhibitors as observed for the previously SAR-derived hnRNP A1 inhibitor IRES-J007. Experiments investigating the mechanism of action of riluzole supported a direct interaction with hnRNP A1 and inhibition of IRES RNA binding. Our working hypothesis is consistent with the notion that these inhibitors likely bind hnRNP A1 within a small pocket which is in close proximity to RRM2 resulting in a conformational rearrangement that precludes IRES binding.

Riluzole is an FDA-approved drug for the treatment of amyotrophic lateral sclerosis (ALS) and several off-label indications for psychiatric and neurologic disorders have been described [29,30,31,32]. Interestingly, pathogenic mutations of hnRNP A1 have been linked to inclusion body formation in ALS [33] and the discovery that riluzole interacts with hnRNP A1 suggests a mechanism for action for this drug in ALS which has thus far been lacking. Riluzole has also recently been shown to exhibit anti-tumor effects in several cancers including gliomas [34,35,36,37,38]. Riluzole possesses glutamatergic modulating and neuroprotective properties, although the precise mechanisms of these effects have not been fully defined [39,40,41]. In fact, the anti-neoplastic effects of riluzole have been reported to be independent of glutamate receptor-1 function in breast cancers [42,43]. Riluzole has additionally been demonstrated to efficiently cross the blood brain barrier and is of significant clinical relevance particularly for the treatment of CNS tumors [44,45].

Our experiments support the direct binding of riluzole to hnRNP A1, which blocks subsequent IRES RNA binding. Our docking studies suggest that riluzole, IRES-J007 and the parent compound C11 bind to a small pocket within close proximity to RRM2. The predicted interacting residues comprising the pocket are highly conserved and appear to form a unique pocket structure. We attempted to superimpose the pocket structure on other known pocket structures to identify possible structural domains that may be similar between targets; however, we were unable to identify any known structural similarities [24]. While this suggests that the pocket is distinct and less likely to exhibit off-target effects, the observation that riluzole can bind this pocket does suggest some degree of binding pocket promiscuity [46].

The flavonoid quercetin has been shown to restrict prostate cancer cell growth and bind directly to hnRNP A1 [47]. Quercetin was shown to bind the F-peptide region of hnRNP A1 blocking its ability to shuttle between the nucleus and cytoplasm resulting in cytoplasmic retention. Redistribution of hnRNP A1 correlated with reduced binding of hnRNP A1 to a requisite nuclear import factor, Tnpo1, consistent with a defect in nucleo-cytoplasmic shuttling [48,49]. The accumulation of hnRNP A1 in the cytoplasm was suggested to inhibit IRES-mediated translation of antiapoptotic mRNAs triggering apoptosis. Riluzole, IRES-J007 and C11 inhibit hnRNP A1 binding to cyclin D1 and c-MYC IRES RNAs and appear to act by a different mechanism then quercetin. Further structural studies are required to provide clear insight into these interactions.

Several potent small-molecule inhibitors capable of specifically inhibiting viral (HCV, EMCV, polio virus) and cellular IRESs have been identified [22,50,51]. The precise mechanism of action of many of these inhibitors is unclear. It has been hypothesized that these agents may function by intercalating into IRES RNA structures and blocking binding of the 40S ribosomal subunit [52]. The degree of IRES inhibition differs significantly between these inhibitors and is likely due to the structural complexity of the interactions between the IRES and the drug. C11, IRES-J007 and riluzole target a specific ITAF-RNA interaction and may exhibit greater specificity then current intercalating agents.

Our data demonstrate that riluzole can display synergistic anti-GBM effects with mTOR inhibitors (see Figure 5C) and recent data have demonstrated that riluzole synergizes with the chemotherapeutic paclitaxel in triple-negative breast cancers [53]. It is well known that chemotherapeutically induced cellular stress can activate IRES-mediated protein synthesis of various transcripts, including the aryl hydrocarbon receptor, ATF2, ribosome binding protein 1, c-myc and *β*-catenin [54,55,56,57,58]. While c-myc IRES-dependent translation is mediated via hnRNP A1, it is tempting to speculate that these other IRES containing mRNAs may also be regulated via hnRNP A1 activity and that these compounds may be utilized to identify other transcripts subject to hnRNP A1-mediated IRES regulation.

In conclusion, we have identified riluzole as an inhibitor of cyclin D1 and c-myc IRES activity. Riluzole was shown to directly bind to hnRNP A1 and abrogate cyclin D1 and c-myc IRES RNA binding. Riluzole was demonstrated to synergize with the direct mTOR kinase inhibitor PP242 in vitro and co-therapy experiments in xenografted mice demonstrated a marked reduction in tumor growth. These data support the repurposing of riluzole as a possible therapeutic for GBM when used in combination with mTOR inhibitors and the targeting of alternative modes of translation initiation to overcome resistance to these agents.

## 4. Materials and Methods

### 4.1. Cell Lines, Constructs and Transfections

Glioblastoma lines LN229 and T98G were obtained from ATCC (Manassas, VA, USA) and the short-term PDX line GBM6 was kindly provided by Dr. Jann Sarkaria (PDX National Resource, Translational Neuro-Oncology, Mayo Clinic, Rochester, MN, USA). The IRES reporter constructs pRF, pRCD1F and pRmycF have been described previously [20]. The pGEX-2T/hnRNP A1 (full-length hnRNP A1) and pGEX-2T/UP1 GST fusion constructs were kindly provided by Ronald Hay (Centre for Gene Regulation and Expression, University of Dundee, Dundee, Scotland, UK) and used to generate additional deletion mutants [24]. To generate the hnRNP A1 alanine substitution mutants, the full-length hnRNP A1 containing plasmid was mutagenized using the QuikChange Lightning Site-Directed Mutagenesis kit (Agilent Technologies, Santa Clara, CA, USA) using appropriate mutagenic primers according to the manufacturer. All plasmids were sequenced to verify the constructs. DNA transfections were performed using Effectene transfection reagent according to the manufacturer (Qiagen, Valencia, CA, USA).

### 4.2. In Silico Docking Screening

For ligand library establishment, 1500 FDA-approved drugs were compiled based on DrugBank 4.0 (http://www.drugbank.ca) [59]. For receptor preparation, the steric structure of monomeric UP1 was derived from the crystal structure deposited in the RCSB PDB (1HA1). The UP1 structure was pre-processed and hydrogen atoms were added prior to docking simulations. Docking was performed using AutoDock Vina [25] and models were visualized using PyMOL v1.5.6 (Schrödinger, LLC, San Diego, CA, USA).

### 4.3. Recombinant Proteins, Antibodies and Reagents

Recombinant native and mutant hnRNP A1 was expressed and purified from HEK293 cells using anti-glutathione Sepharose column chromatography as previously described [20]. Antibodies were from the following sources: mouse IgG (Santa Cruz Biotechnology, Santa Cruz, CA, USA), hnRNP A1 (Abcam, Burlingame, CA, USA), actin (Abcam), cyclin D1 (Cell Signaling Technology, Danvers, MA, USA), c-MYC (Cell Signaling Technology), anti-GST (Cell Signaling Technology). PP242 and rapamycin were obtained from LC Laboratories (Woburn, MA, USA). IRES-J007 was synthesized as previously described [24] and all other reagents were from Sigma.

### 4.4. IRES Reporter Assays, In Vitro RNA Pull-Down Assays, Filter Binding Assays and Polysome Analyses

For IRES reporter assays, the indicated mRNA reporters were cotransfected into cells with pSV*β*-galactosidase to normalize for transfection efficiency as described previously [18]. Cells were harvested 18 h following transfection and *Renilla*, firefly and β-galactosidase activities determined using the Dual-Glo^®^ Luciferase and β-galactosidase assay systems (Promega, Madison, WI, USA). For RNA pull-down assays [20], cytoplasmic extracts were prepared by hypotonic lysis in buffer containing 10 mM HEPES (pH 7.5), 10 mM potassium acetate, 1.5 mM magnesium acetate, 2.5 mM DTT, 0.05% NP-40, 10 mM NaF, 1 mM sodium orthovanidate, 1 mM PMSF and 1.5% aprotinin using a Dounce homogenizer. Extracts were precleared by centrifugation, and SUPERase-IN (ThermoFisher, 0.025 units/mL) and yeast tRNA (15 µg/mL) were added and applied to an equilibrated heparin-agarose column (Bio-Rad). Eluates were further cleared with 100 µL of streptavidin-Sepharose (Sigma, St. Louis, MO, USA) for 1 h at 4 °C. Following centrifugation, 10 µg of in vitro transcribed biotinylated IRES RNA (mMESSAGE Machine T7 transcription kit, ThermoFisher, Waltham, MA, USA) was added to the supernatant and incubated for 1 h at 4 °C. The protein and biotinylated RNA complexes were recovered by adding 30 µL of streptavidin-Sepharose, which was incubated for 2 h at 4 °C. The complexes were washed five times in binding buffer (10 mM HEPES (pH 7.5), 90 mM potassium phosphate, 1.5 mM magnesium acetate, 2.5 mM DTT, 0.05% NP-40, 10 mM NaF, 1 mM sodium orthovanidate, 1 mM PMSF and 1.5% aprotinin) and then boiled in SDS and resolved by gel electrophoresis and immunoblotted. Filter binding assays were performed as previously described [20,60]. GST-tagged hnRNP A1 or hnRNP A1 deletion mutants were added to in vitro transcribed ^32^P-labeled RNAs corresponding to either the cyclin D1 or c-MYC IRESs in separate reactions in a volume of 10 µL in buffer containing 5 mM HEPES (pH 7.6), 30 mM KCl, 2 mM MgCl_2_, 200 mM DTT, 4% glycerol and 10 ng yeast tRNA for 10 min at room temperature. 8 µL of each binding reaction was applied to nitrocellulose membranes on a slot blot apparatus (Minifold II, Schleicher & Schuell, Keene, NH, USA). Membranes were washed and dried, and signals quantified using a phosphorimager. Separation of polysomes was performed as previously described [26]. Briefly, cell extracts were prepared and layered onto 15% to 50% sucrose gradients and spun at 38,000 rpm for 2 h at 4 °C in a SW40 rotor (Beckman Instruments, Brea, CA, USA). Gradients were fractionated using a gradient fractionator system (Brandel Instruments, Gaithersburg, MD, USA) using a flow rate of 3 mL/min. The polysome profile of the gradients was monitored via UV absorbance at 260 nm. RNA was isolated and pooled into nonpolysomal and polysomal fractions. RNAs (100 ng) were subsequently used in quantitative reverse transcriptase-PCR analyses.

### 4.5. Immunoblotting and Quantitative Real-Time PCR

Western blotting was performed as previously described [26]. miRNeasy mini kit (Qiagen) was used to isolate total RNA and was reverse transcribed into cDNA using the High Capacity RNA-to-cDNA kit (ABI). Taqman primers (from Applied Biosystems, Waltham, MA, USA) were used to measure the levels of all transcripts and analyses were performed on an ABI7900HT system (ABI ThermoFisher, Waltham, MA, USA).

### 4.6. Cell Proliferation, Cell-Cycle Distribution and TUNEL Assays

Cell proliferation was determined via Cell Titer-Glo^®^ luminescent cell assays (Promega) and cell-cycle analysis was done by propidium iodide staining of cells and flow cytometry as previously described [61]. Cells were stained using a FITC-conjugated annexin V (Annexin V-FITC Early Apoptosis Detection kit, Cell Signaling Technology) to monitor apoptosis. TUNEL staining of tumor sections was performed using the TACSXL DAB In Situ Apoptosis Detection kit (Trevigen, Gaithersburg, MD, USA) according to the manufacturer’s instructions [26]. The combination index (CI) values were determined by using CalcuSyn v2.0 software (Biosoft, Cambridge, UK) [26].

### 4.7. Xenograft Studies

All animal experiments were performed under an approved Institutional Animal Care and Use Committee protocol (Greater Los Angeles VA Healthcare System, Institutional Animal Care and Use Committee, protocol # 01002-14) (approved 15- January-2017) and conformed to the guidelines established by the Association for the Assessment and Accreditation of Laboratory Animal Care. Xenografts of LN229 cells were performed in female C.B.-17-scid (Taconic, Germantown, NY, USA) mice as previously described [16,26]. Tumors were harvested at autopsy for immunoblot blot analysis. Sections of paraffin-embedded tumors on slides were processed for immunohistochemistry as previously described [26].

### 4.8. Statistical Analyses

Statistical analyses were performed with Student’s *t*-test and ANOVA models using Systat 13 (Systat Software, Chicago, IL, USA). *p*-values of less then 0.05 were considered significant.

## Supplementary Materials

Supplementary materials can be found at https://www.mdpi.com/1422-0067/21/1/344/s1. Supplementary materials include Table S1, Table S2 and Figure S1.

## Abbreviations

IRES	Internal ribosome entry site
ITAF	IRES trans-acting factor
ALS	Amyotrophic lateral sclerosis
hnRNP A1	Heterogeneous nuclear ribonucleoprotein A1
mTOR	Mechanistic target of rapamycin
mTORC	Mechanistic target of rapamycin complex
CNS	Central nervous system
PI3K	Phosphatidylinositol 3-kinase
EGFR	Epidermal growth factor receptor
PTEN	Phosphatase and tensin homolog
AKT/PKB	Protein kinase B
SPR	Surface plasmon resonance
FDA	Food and drug administration, United States
PDX	Patient derived xenograft
RNP	Ribonucleoprotein
RRM	RNA recognition motif
GST	Glutathione S-transferase
FITC	Fluorescein isothiocyanate
SCID	Severe combined immunodeficient
TUNEL	Terminal deoxynucleotidyl transferase dUTP nick end labeling
qrt-PCR	Quantitative reverse transcription polymerase chain reaction
