# Supplementary material for: Repurposing Potential of Riluzole as an ITAF Inhibitor in mTOR Therapy Resistant Glioblastoma
Source: Int J Mol Sci. 2020 Jan 5;21(1):344. doi: 10.3390/ijms21010344 (PMC6981868; doi:10.3390/ijms21010344)
Supplement: Supplementary file 1 [file ijms-21-00344-s001.zip › TUNEL riluzole supplementary figure S1.pptx.pdf]

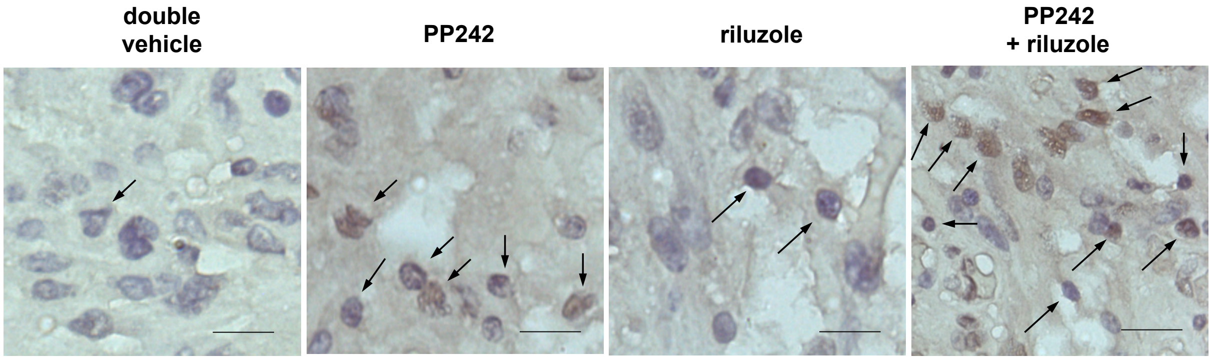


**Figure S1.** Representative TUNEL assay results from LN229 tumor xenografts receiving the indicated therapies. As shown only rare apoptotic (brown) signals in monotherapy-treated animals and significantly greater numbers of apoptotic cells in combination therapy-treated mice. Scale bar, 20 µm.
